# S100A4 Is Involved in Stimulatory Effects Elicited by the FGF2/FGFR1 Signaling Pathway in Triple-Negative Breast Cancer (TNBC) Cells

**DOI:** 10.3390/ijms22094720

**Published:** 2021-04-29

**Authors:** Maria Francesca Santolla, Marianna Talia, Marcello Maggiolini

**Affiliations:** Department of Pharmacy, Health and Nutritional Sciences, University of Calabria, 87036 Rende, Italy; mariafrancesca.santolla@unical.it (M.F.S.); marianna.talia@unical.it (M.T.)

**Keywords:** TNBC, FGF2, FGFR1, S100A4, tumor angiogenesis, CAFs

## Abstract

Triple-negative breast cancer (TNBC) is an aggressive breast tumor subtype characterized by poor clinical outcome. In recent years, numerous advancements have been made to better understand the biological landscape of TNBC, though appropriate targets still remain to be determined. In the present study, we have determined that the expression levels of FGF2 and S100A4 are higher in TNBC with respect to non-TNBC patients when analyzing “The Invasive Breast Cancer Cohort of The Cancer Genome Atlas” (TCGA) dataset. In addition, we have found that the gene expression of FGF2 is positively correlated with S100A4 in TNBC samples. Performing quantitative PCR, Western blot, CRISPR/Cas9 genome editing, promoter studies, immunofluorescence analysis, subcellular fractionation studies, and ChIP assays, we have also demonstrated that FGF2 induces in TNBC cells the upregulation and secretion of S100A4 via FGFR1, along with the ERK1/2–AKT–c-Rel transduction signaling. Using conditioned medium from TNBC cells stimulated with FGF2, we have also ascertained that the paracrine activation of the S100A4/RAGE pathway triggers angiogenic effects in vascular endothelial cells (HUVECs) and promotes the migration of cancer-associated fibroblasts (CAFs). Collectively, our data provide novel insights into the action of the FGF2/FGFR1 axis through S100A4 toward stimulatory effects elicited in TNBC cells.

## 1. Introduction

Breast cancer is the most frequently diagnosed tumor and represents the second leading cause of cancer death among females worldwide [1]. To date, it has been identified different subgroups of breast cancers with peculiar prognosis and response to chemotherapeutics [2,3,4,5,6,7]. For instance, triple-negative breast cancer (TNBC), characterized by the absence of the estrogen receptor (ER), progesterone receptor (PR), and epidermal growth factor receptor 2 (HER2) [8,9], is associated with a high risk of recurrence and poor prognosis [10,11]. Currently, the key limiting factor in the treatment of TNBC is the lack of tailored therapeutic strategies. Hence, in the last years, great efforts have been made toward the identification of signaling pathways contributing to the TNBC progression [12,13,14]. In this regard, fibroblast growth factor (FGF)/FGF receptor (FGFR) axis has been indicated as a potential candidate for targeted therapy [15,16,17]. FGFRs are receptor tyrosine kinases which encompass four key members (FGFR1 to FGFR4) and one receptor that binds to FGF ligands, although it lacks the intracellular kinase domain (FGFR5, also named FGFRL1) [18,19,20]. FGFRs are involved in diverse pathophysiological conditions and cell responses such as survival, growth, motility, apoptosis, and angiogenesis [21,22,23,24]. It is worth mentioning that TNBC cells may show genomic FGFR alterations along with an altered FGF2 signaling loop, suggesting that FGFR inhibitors and monoclonal antibodies could be considered in comprehensive therapeutic approaches aimed to halt TNBC progression [25,26].

Increasing evidence has demonstrated that the tumor microenvironment mainly contributes to cancer development and metastatic dissemination [27]. For instance, diverse components of the tumor stroma may influence the spread of tumor cells, providing certain signals that trigger invasive phenotypes [28,29]. In this regard, the S100 calcium-binding protein A4 (S100A4) that belongs to the S100 family proteins has been shown to contribute to the functional liaison between cancer cells and the surrounding microenvironment toward worse outcomes [30,31,32,33,34,35,36]. Corroborating these findings, an increased level of S100A4 has been detected in breast cancer interstitial fluid and in the serum of S100A4 transgenic mice [37,38]. Furthermore, it has been demonstrated that S100A4 secreted by either tumor and/or stromal cells may lead to pro-metastatic events such as cell motility, invasion, and angiogenesis [39]. Indeed, extracellular S100A4, acting in a paracrine manner, stimulates biological responses through the interaction with the receptor for advanced glycation end products (RAGE) [40,41,42,43]. RAGE, which is a multi-ligand transmembrane receptor belonging to the immunoglobulin superfamily, may be involved in several clinical disorders, including cancer [44,45]. Furthermore, RAGE activation may induce proliferative, angiogenic, migratory, and inflammatory effects associated with a poor prognosis in several tumors such as breast cancer [46,47,48,49].

Here, performing a bioinformatics analysis on the publicly available TCGA dataset, we have ascertained that FGF2 expression is positively correlated with S100A4 in a large cohort of TNBC patients. Moreover, we have assessed the molecular mechanisms through which the FGF2/FGFR1 axis triggers S100A4 expression and secretion, leading to important biological responses that may contribute to TNBC progression.

## 2. Results

### 2.1. The Gene Expression Levels of FGF2 and S100A4 Are Correlated in TNBC Patients

**Figure 1:**
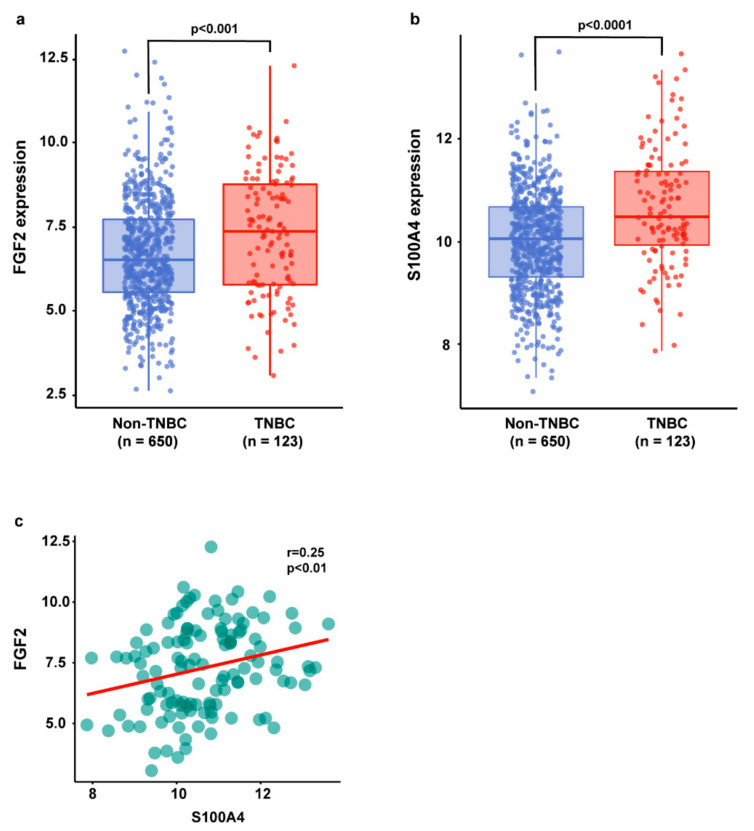
FGF2 and S100A4 gene expression levels in TCGA dataset. Box plots showing the differential expression of FGF2 (**a**) and S100A4 (**b**) in non-TNBC and TNBC patients. (**c**) Scatter plot depicting the correlation between the expression of FGF2 and S100A4 in TNBC samples of the TCGA cohort. The number of patients and *p*-values are reported in each panel.

Previous studies have shown that diverse stimuli, including growth factors, regulate S100A4 expression in cancer cells toward aggressive tumor features [36,50,51,52,53,54]. As it concerns TNBC, the overexpression of S100A4 has been reported to promote cell motility, invasion, and metastasis [55]. Furthermore, several reports have also highlighted a significant correlation between the expression of S100A4 and advanced tumor stage, the presence of distant metastases, and worse survival rates in breast cancer patients [36,56,57,58]. On the basis of these findings, we began our investigation by querying “The Invasive Breast Cancer Cohort of The Cancer Genome Atlas” (TCGA) dataset, which supplies RNA-Seq data and clinical information of breast cancer patients. Of note, the FGF2 and S100A4 gene expression levels were found to be higher in TNBC with respect to non-TNBC patients (Figure 1a,b). In addition, a positive correlation between FGF2 and S100A4 levels was assessed in TNBC samples (Figure 1c), suggesting their potential cooperation in this aggressive breast cancer subtype.

### 2.2. FGF2/FGFR1 Mediated Signaling Upregulates S100A4 Levels in TNBC Cells

**Figure 2:**
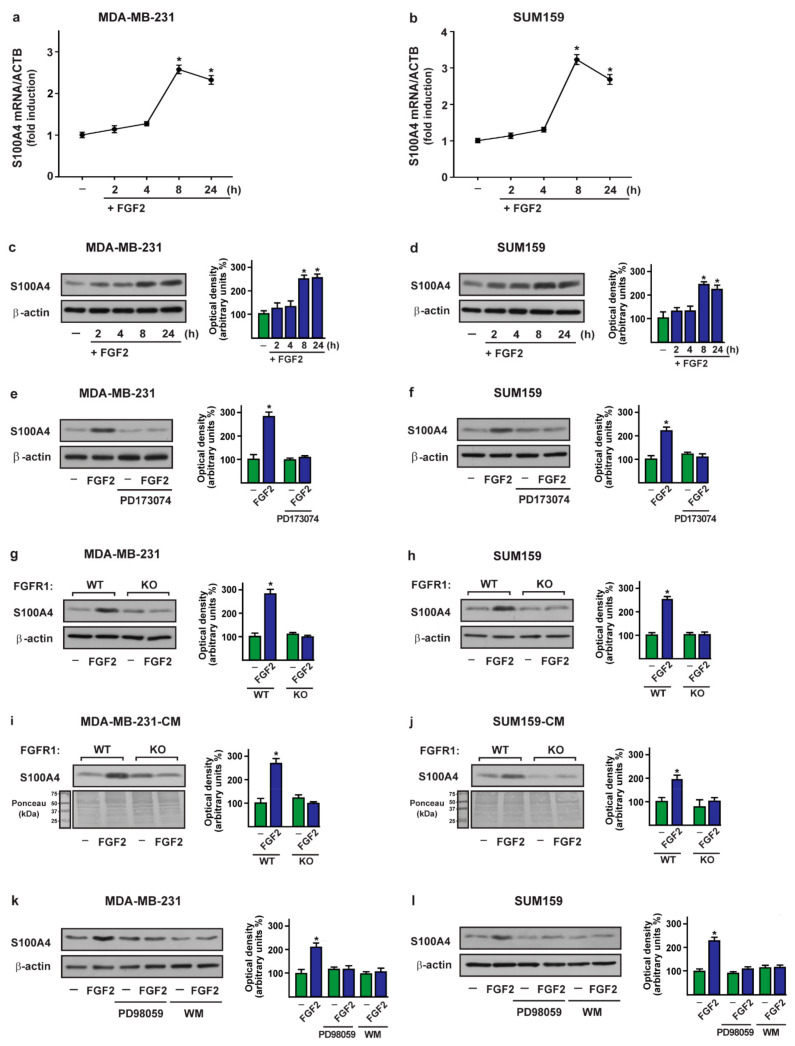
The FGF2/FGFR1 transduction pathway upregulates S100A4 expression in MDA-MB-231 and SUM159 cells. mRNA (**a**,**b**) and protein (**c**,**d**) expression of S100A4 evaluated respectively by real-time PCR and immunoblotting in MDA-MB-231 and SUM159 cells treated with vehicle (-) and 25 nM FGF2, as indicated. In RNA experiments, values were normalized to the β-actin (ACTB) expression and shown as fold changes of S100A4 mRNA expression upon FGF2 treatment compared to cells exposed to vehicle (-). (**e**,**f**) Immunoblots showing S100A4 protein expression in MDA-MB-231 and SUM159 cells exposed for 8 h to 25 nM FGF2 alone and in combination with 1 µM FGFR1 inhibitor PD173074. (**g**,**h**) S100A4 protein expression evaluated by immunoblotting in FGFR1 (WT) and FGFR1 (KO) MDA-MB-231 and SUM159 cells treated for 8 h with vehicle (-) and 25 nM FGF2, as indicated. (**i**,**j**) Evaluation by immunoblotting of S100A4 levels in conditioned medium (CM) collected from FGFR1 (WT) and FGFR1 (KO) MDA-MB-231 and SUM159 cells treated for 8 h with vehicle (-) and 25 nM FGF2. Ponceau red staining of the membrane was used as a loading control for the CM. (**k**,**l**) Immunoblots showing S100A4 protein expression in MDA-MB-231 and SUM159 cells exposed for 8 h to vehicle (-) and 25 nM FGF2 alone and in the presence of 1 µM MEK inhibitor PD98059 or 100 nM PI3K inhibitor Wortmannin (WM). β-Actin served as a loading control. Side panels show densitometric analysis of the blots normalized to the loading control. Values represent the mean ± SD of three independent experiments. (*) indicates *p* < 0.05.

Then, we aimed to evaluate whether FGF2 may regulate S100A4 levels in MDA-MB-231 and SUM159 cells that were used as a model system of TNBC. Of note, FGF2 induced the expression of S100A4 at both mRNA (Figure 2a,b) and protein levels in TNBC cells (Figure 2c,d). However, the S100A4 protein expression upon FGF2 exposure was no longer evident using the FGFR1 inhibitor PD173074 (Figure 2e,f) and in FGFR1 knockout (KO) MDA-MB-231 and SUM159 cells (Figure 2g,h and Supplementary Figure S1a,b), which were obtained by CRISPR/Cas9-mediated genome editing. Next, the secretion of S100A4 upon treatment with FGF2 was evaluated in conditioned medium collected from FGFR1 (WT) and FGFR1 (KO) MDA-MB-231 and SUM159 cells. S100A4 levels were found upregulated by FGF2 in conditioned medium derived from FGFR1 (WT) MDA-MB-231 and SUM159 cells, but not in conditioned medium derived from FGFR1 (KO) MDA-MB-231 and SUM159 cells treated with FGF2 (Figure 2i,j). Considering that FGFR1 activation by FGF2 triggers certain transduction pathways [59,60], we assessed that the upregulation of S100A4 induced by FGF2 is prevented using the MEK inhibitor PD98059 and the PI3K inhibitor Wortmannin (WM) (Figure 2k,l), but not in the presence of the STAT3 inhibitor STA21 and the JNK inhibitor SP600125 (SP) (data not shown).

**Figure 3:**
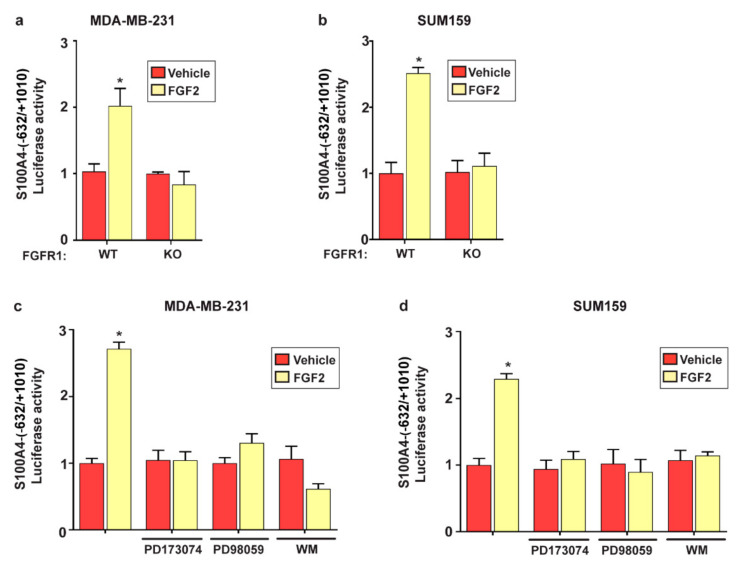
The FGF2/FGFR1-mediated signaling activates the S100A4 promoter construct. (**a**,**b**) Luciferase activities of the S100A4 promoter construct in FGFR1 (WT) and FGFR1 (KO) MDA-MB-231 and SUM159 cells treated for 18 h with vehicle and 25 nM FGF2. (**c**,**d**) Luciferase activities of the S100A4 promoter construct in MDA-MB-231 and SUM159 cells treated for 18 h with vehicle and 25 nM FGF2 alone and in combination with 1 µM FGFR1 inhibitor PD173074, 1 µM MEK inhibitor PD98059, or 100 nM PI3K inhibitor Wortmannin (WM), as indicated. The luciferase activities were normalized to the internal transfection control and values of cells receiving vehicle were set as 1-fold induction upon which the activities induced by treatments were calculated. Each column represents the mean ± SD of three independent experiments performed in triplicate. (*) indicates *p* < 0.05.

In support of these findings, the treatment with FGF2 induced the phosphorylation of FGFR1 as well as the activation of ERK1/2 and AKT in MDA-MB-231 and SUM159 cells (Supplementary Figure S2a,b). Moreover, we ascertained that in MDA-MB-231 and SUM159 cells, the activation of ERK1/2 and AKT upon FGF2 treatment is prevented in the presence of the specific inhibitors PD98059 and Wortmannin (WM), respectively (Supplementary Figure S2c,d). Next, the transcriptional activation of the S100A4 promoter construct prompted by FGF2 in FGFR1 (WT) MDA-MB-231 and SUM159 cells was no longer evident in FGFR1 (KO) MDA-MB-231 and SUM159 cells (Figure 3a,b) or using the FGFR1 inhibitor PD173074, the MEK inhibitor PD98059, and the PI3K inhibitor Wortmannin (WM) in FGFR1 (WT) MDA-MB-231 and SUM159 cells (Figure 3c,d). Taken together, these findings indicate that FGF2 induces S100A4 expression through the activation of the FGFR1–ERK1/2–AKT transduction pathway in TNBC cells.

### 2.3. c-Rel Is Involved in the Upregulation of S100A4 Induced by FGF2/FGFR1 Signaling

**Figure 4:**
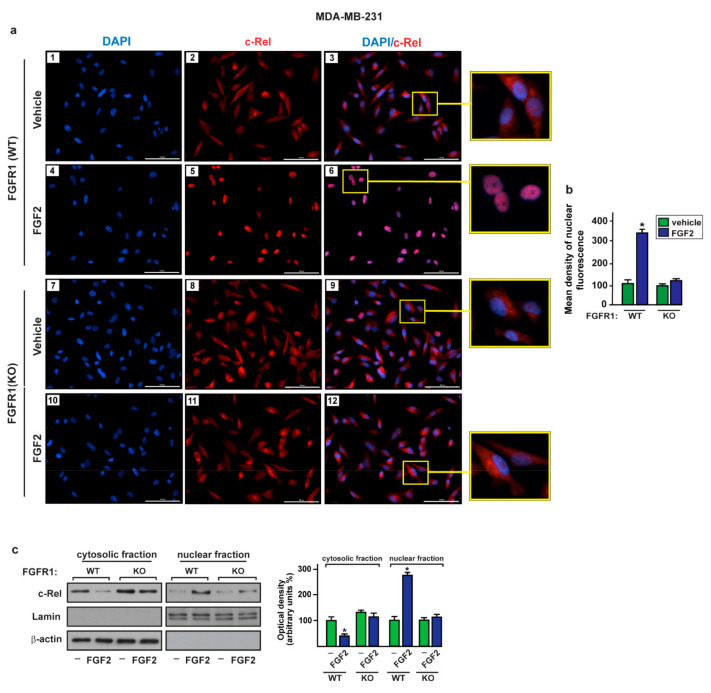
The activation of FGF2/FGFR1 transduction pathway leads to c-Rel nuclear accumulation in MDA-MB-231 cells. (**a**) Immunofluorescence staining of c-Rel in FGFR1 (WT) (panels 1–6) and FGFR1 (KO) (panels 7–12) MDA-MB-231 cells treated for 2 h with vehicle and 25 nM FGF2. c-Rel localization is shown in red, nuclei are stained by DAPI (blue), scale bar = 100 µm. Enlarged details are shown in the separate box. Images shown are representative of two independent experiments. (**b**) Quantification of nuclear c-Rel in cells treated with vehicle versus cells exposed to FGF2, as indicated. (**c**) Immunoblots of cytosolic and nuclear fraction lysates derived from FGFR1 (WT) and FGFR1 (KO) MDA-MB-231 cells treated for 2 h with vehicle (-) and 25 nM FGF2, as indicated. β-Actin and lamin were used as cytosolic and nuclear protein markers, respectively. Side panel shows densitometric analysis of the blots normalized to actin (for cytosolic fraction) or to lamin (for nuclear fraction). Values represent the mean ± SD of three independent experiments. (*) indicates *p* < 0.05.

**Figure 5:**
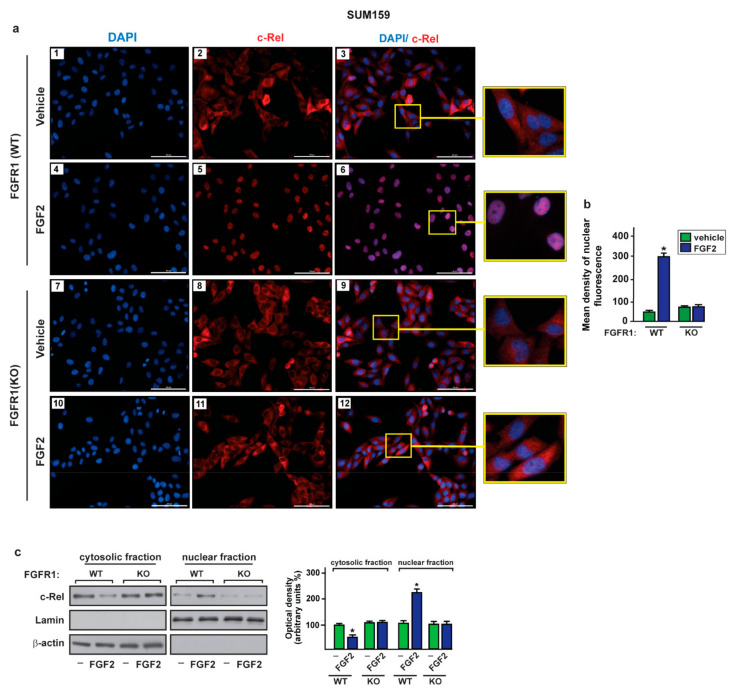
The activation of FGF2/FGFR1 transduction pathway leads to c-Rel nuclear accumulation in SUM159 cells. (**a**) Immunofluorescence staining of c-Rel in FGFR1 (WT) (panels 1–6) and FGFR1 (KO) (panels 7–12) SUM159 cells treated for 2 h with vehicle and 25 nM FGF2. c-Rel localization is shown in red, nuclei are stained by DAPI (blue), scale bar = 100 µm. Enlarged details are shown in the separate box. Images shown are representative of two independent experiments. (**b**) Quantification of nuclear c-Rel in cells treated with vehicle versus cells exposed to FGF2, as indicated. (**c**) Immunoblots of cytosolic and nuclear fraction lysates derived from FGFR1 (WT) and FGFR1 (KO) SUM159 cells treated for 2 h with vehicle (-) and 25 nM FGF2, as indicated. β-Actin and lamin were used as cytosolic and nuclear protein markers, respectively. Side panel shows densitometric analysis of the blots normalized to actin (for cytosolic fraction) or to lamin (for nuclear fraction). Values represent the mean ± SD of three independent experiments. (*) indicates *p* < 0.05.

It has been reported that FGFR1 activation triggers the NF-kB signaling cascade in diverse cell contexts, including breast tumor cells [61,62,63,64]. The NF-kB subunit named c-Rel was indicated to be a transcription factor involved in NF-kB signaling in breast cancer [65,66,67]. In order to investigate whether c-Rel may be engaged in the upregulation of S100A4 prompted by the FGF2/FGFR1 axis, we ascertained that the exposure to FGF2 induces the nuclear shuttle of c-Rel in FGFR1 (WT) MDA-MB-231 and SUM159 cells but not in FGFR1 (KO) MDA-MB-231 and SUM159 cells, as demonstrated by immunofluorescence (Figure 4a,b and Figure 5a,b) and subcellular fractionation studies (Figure 4c and Figure 5c).

**Figure 6:**
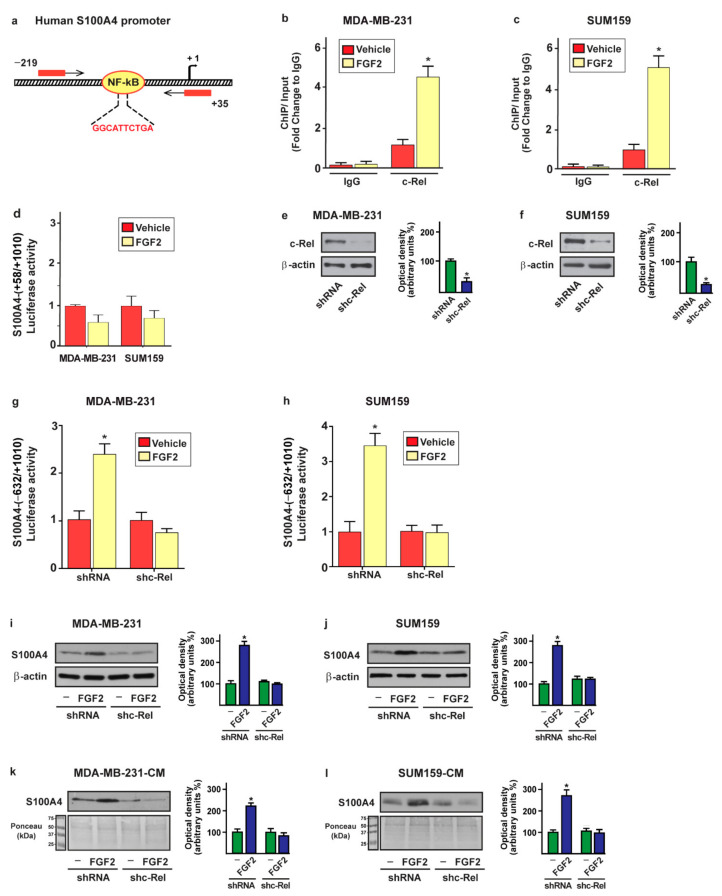
c-Rel is involved in the upregulation of S100A4 levels prompted by FGF2 in MDA-MB-231 and SUM159 cells. (**a**) Schematic representation of human S100A4 promoter carrying the NF-kB-responsive site (the transcriptional start site is indicated as + 1). (**b**,**c**) The treatment for 2 h with 25 nM FGF2 induces the recruitment of c-Rel to the NF-kB site located within the S100A4 promoter region in MDA-MB-231 and SUM159 cells, as ascertained by chromatin immunoprecipitation (ChIP) assay. Data were normalized to the input and reported as fold changes with respect to IgG. Each column represents the mean ± SD of three independent experiments performed in triplicate. (**d**) Luciferase activities of the S100A4 promoter construct in MDA-MB-231 and SUM159 cells treated for 18 h with vehicle and 25 nM FGF2. (**e**,**f**) Efficiency of c-Rel silencing in MDA-MB-231 and SUM159 cells. (**g**,**h**) Luciferase activities of the S100A4 promoter construct in MDA-MB-231 and SUM159 cells transfected with shRNA or shc-Rel and treated for 18 h with vehicle and 25 nM FGF2. The luciferase activities were normalized to the internal transfection control and values of cells receiving vehicle were set as 1-fold induction upon which the activity induced by treatment was calculated. Each column represents the mean ± SD of three independent experiments performed in triplicate. (**i**,**j**) S100A4 protein expression evaluated by immunoblotting in MDA-MB-231 and SUM159 cells transfected with shRNA or shc-Rel and treated for 8 h with vehicle (-) and 25 nM FGF2. β-Actin served as a loading control. (**k**,**l**) Immunoblotting of S100A4 in conditioned medium (CM) collected from MDA-MB-231 and SUM159 cells transfected with shRNA or shc-Rel and treated for 8 h with vehicle (-) and 25 nM FGF2. Ponceau red staining of the membrane was used as a loading control for the CM. Side panels show densitometric analysis of the blots normalized to the loading control. Values represent the mean ± SD of three independent experiments. (*) indicates *p* < 0.05.

Through immunofluorescence assays performed in MDA-MB-231 (Supplementary Figure S3a,b) and SUM159 (Supplementary Figure S4a,b) cells, we also determined that c-Rel nuclear translocation upon FGF2 exposure is abrogated in the presence of the FGFR1 inhibitor PD173074, the MEK inhibitor PD98059 or the PI3K inhibitor Wortmannin (WM). However, whole c-Rel protein levels were not altered upon treatment with FGF2 in both MDA-MB-231 and SUM159 cells (Supplementary Figure S5a–d). Thereafter, we assessed that FGF2 treatment induces the recruitment of c-Rel to the NF-kB site located within the S100A4 promoter sequence in MDA-MB-231 and SUM159 cells, as demonstrated by chromatin immunoprecipitation (ChIP) assay (Figure 6a–c). Further supporting these results, the treatment with FGF2 did not induce transactivation of the S100A4 promoter construct, lacking the NF-kB binding site in both MDA-MB-231 and SUM159 cells (Figure 6d). Remarkably, the silencing of c-Rel abolished the transcriptional activation of the S100A4 promoter construct upon FGF2 in MDA-MB-231 and SUM159 cells (Figure 6e–h). In accordance with these findings, the S100A4 protein induction (Figure 6i,j) and secretion (Figure 6k,l) triggered by FGF2 prevented silencing c-Rel in MDA-MB-231 and SUM159 cells. Collectively, these results suggest that activation of the FGFR1–ERK1/2–AKT–c-Rel signaling pathway mediates the upregulation of S100A4 levels in both MDA-MB-231 and SUM159 cells.

### 2.4. The Paracrine Activation of S100A4/RAGE Signaling Induces Endothelial Tube Formation in HUVECs

**Figure 7:**
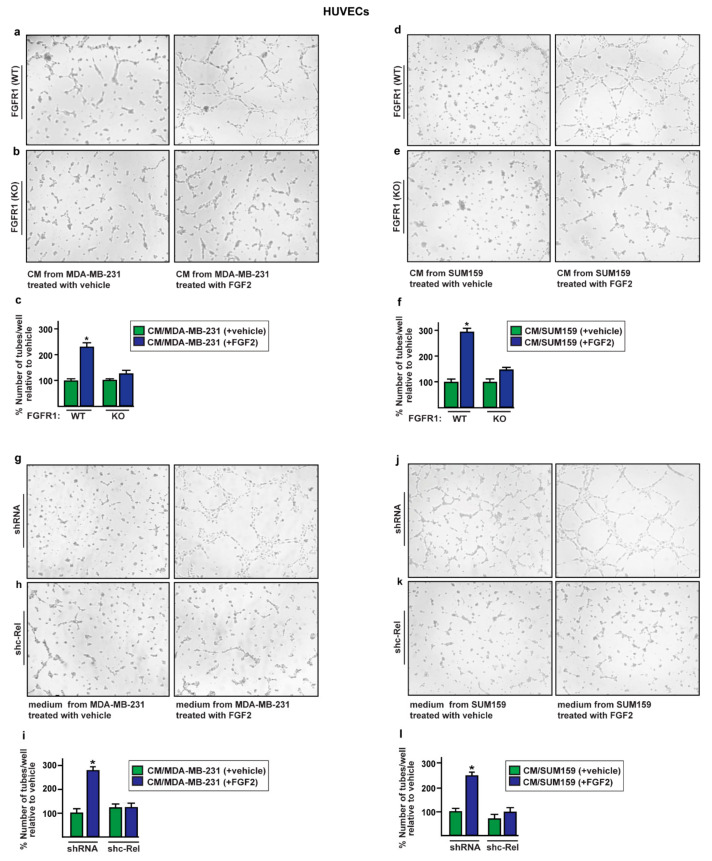
Conditioned medium (CM) from FGF2-stimulated MDA-MB-231 and SUM159 cells triggers endothelial tube formation. (**a**,**b**,**d**,**e**) Tube formation evaluated in HUVECs cultured for 6 h in CM collected from FGFR1 (WT) and FGFR1 (KO) MDA-MB-231 and SUM159 cells treated for 8 h with vehicle and 25 nM FGF2. Tube formation in HUVECs cultured for 6 h in CM collected from MDA-MB-231 and SUM159 cells transfected with shRNA (**g**,**h**) or shc-Rel (**j**,**k**) and then treated for 8 h with vehicle and 25 nM FGF2. (**c**,**f**,**i**,**l**) Quantification of the number of tubes observed in HUVECs, as indicated. Data are representative of three independent experiments performed in triplicate. (*) indicates *p* < 0.05.

S100A4 released by tumor and stromal cells within the tumor microenvironment may contribute to cell proliferation, invasion, angiogenesis, and metastasis [34,38,42,56,58,68]. In this regard, previous studies have demonstrated that several S100 family members, including S100A4, act in both an autocrine and paracrine manner mainly binding to and activating RAGE-mediated signaling in different cell types [42,69,70]. On the basis of these observations, we aimed to evaluate the paracrine effects of S100A4 derived from FGF2-stimulated MDA-MB-231 and SUM159 cells on vascular endothelial cells (HUVECs). Hence, conditioned medium from MDA-MB-231 and SUM159 cells exposed to FGF2 was collected and used as culture medium in HUVECs in order to perform tube formation assay. Upon this experimental condition, HUVECs showed a complex and ramified network of tubules (Figure 7a,d), while no tube formation was observed using conditioned medium from FGFR1 (KO) MDA-MB-231 and SUM159 cells exposed to FGF2 (Figure 7b,e) or in the presence of the FGFR1 inhibitor PD173074 (data not shown).

**Figure 8:**
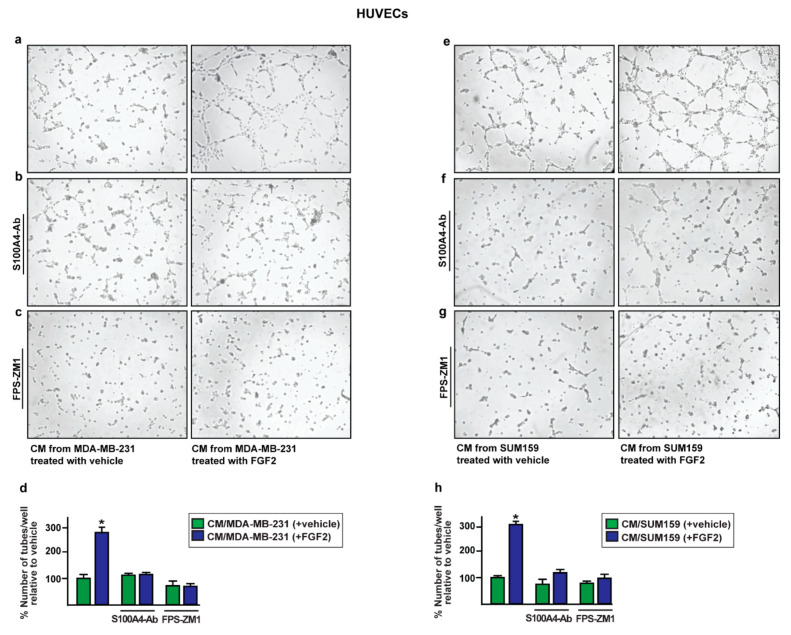
The paracrine activation of S100A4/RAGE signaling induces endothelial tube formation. The endothelial tube formation observed in HUVECs cultured for 6 h in conditioned medium (CM) collected from MDA-MB-231 and SUM159 cells treated for 8 h with 25 nM FGF2 (**a**,**e**), was no longer evident using CM collected from MDA-MB-231 and SUM159 cells which were treated for 8 h with vehicle and 25 nM FGF2 but immunodepleted for S100A4 by incubation with a mouse monoclonal antibody against S100A4 (S100A4-Ab) (**b**,**f**) or adding 2 μM RAGE antagonist FPS-ZM1 (**c**,**g**). (**d**,**h**) Quantification of the number of tubes, detected in HUVECs, as indicated. Data are representative of three independent experiments performed in triplicate. (*) indicates *p* < 0.05.

Similar effects were obtained culturing HUVECs in conditioned medium collected from MDA-MB-231 and SUM159 cells silenced for c-Rel expression and treated with FGF2 (Figure 7g–l). Further corroborating these findings, conditioned medium from MDA-MB-231 and SUM159 cells exposed to FGF2 and immunodepleted for S100A4 (Supplementary Figure S6a,b) reduced the endothelial tube formation of HUVECs as also observed in the presence of the RAGE inhibitor FPS-ZM1 (Figure 8). Altogether, these results suggest that the release of S100A4 from FGF2-stimulated MDA-MB-231 and SUM159 cells promotes the paracrine activation of RAGE signaling toward angiogenic responses.

### 2.5. S100A4/RAGE Paracrine Activation Promotes Cell Migration in Breast Cancer-Associated Fibroblasts (CAFs)

**Figure 9:**
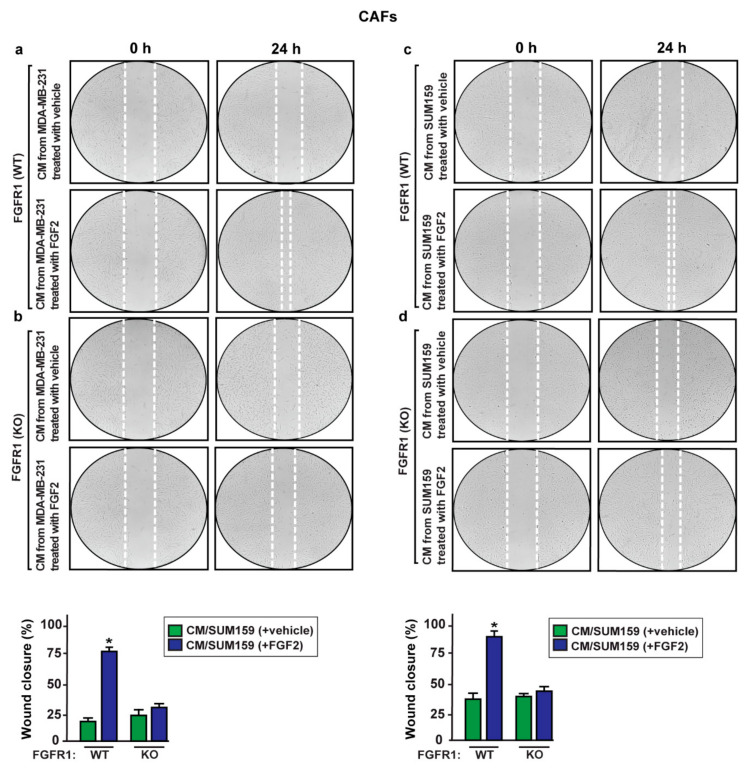
Conditioned medium (CM) from FGF2-stimulated MDA-MB-231 and SUM159 cells induces the migration of CAFs. CAFs were incubated for 24 h with CM collected from FGFR1 (WT) and FGFR1 (KO) MDA-MB-231 (**a**,**b**) and SUM159 (**c**,**d**) cells treated for 8 h with vehicle and 25 nM FGF2. Images were acquired at 0 and 24 h after scratching. Quantification of cell migration was expressed as % of wound closure. Data shown are the mean ± SD of three independent experiments performed in triplicate. (*) indicates *p* < 0.05.

In order to further evaluate the paracrine action exerted by S100A4, we sought to determine whether S100A4 may trigger biological effects on main components of tumor microenvironment as cancer-associated fibroblasts (CAFs). Remarkably, CAFs cultured in conditioned medium collected from MDA-MB-231 and SUM159 cells exposed to FGF2 exhibited an increased migratory capacity (Figure 9a,c) that was absent using the FGFR1 inhibitor PD173074 (data not shown) or when culturing CAFs in conditioned medium from FGFR1 (KO) MDA-MB-231 and SUM159 cells treated with FGF2 (Figure 9b,d).

**Figure 10:**
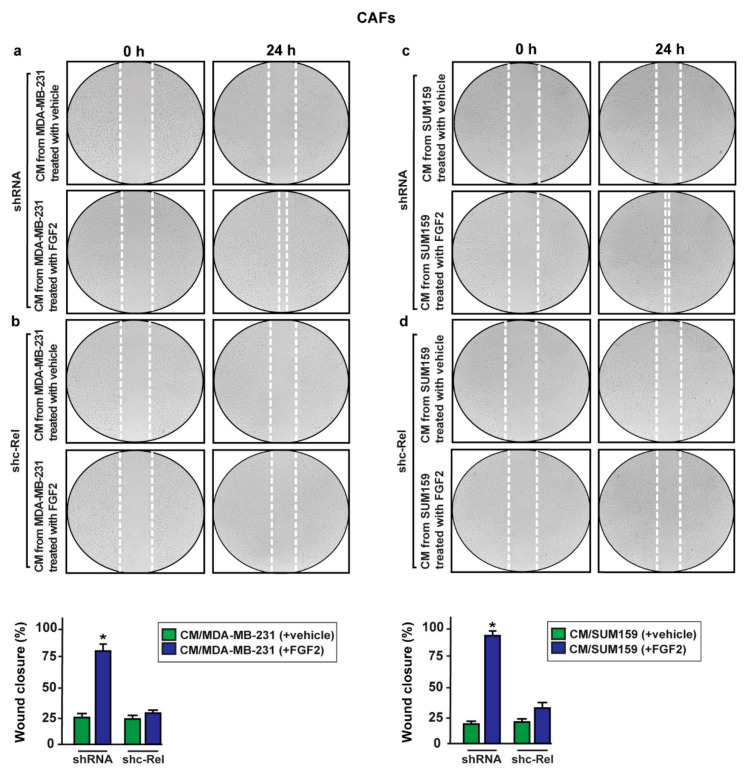
c-Rel is involved in the migration of CAFs triggered by conditioned medium (CM) from FGF2-stimulated MDA-MB-231 and SUM159 cells. CAFs were incubated for 24 h with CM collected from MDA-MB-231 and SUM159 cells transfected with shRNA (**a**,**c**) or shc-Rel (**b**,**d**) and treated for 8 h with vehicle and 25 nM FGF2. Images were acquired at 0 and 24 h after scratching. Quantification of cell migration was expressed as % of wound closure. Data shown are the mean ± SD of three independent experiments performed in triplicate. (*) indicates *p* < 0.05.

Similarly, we did not observe the migration of CAFs cultured with conditioned medium from MDA-MB-231 and SUM159 cells silenced for c-Rel expression and treated with FGF2 (Figure 10).

**Figure 11:**
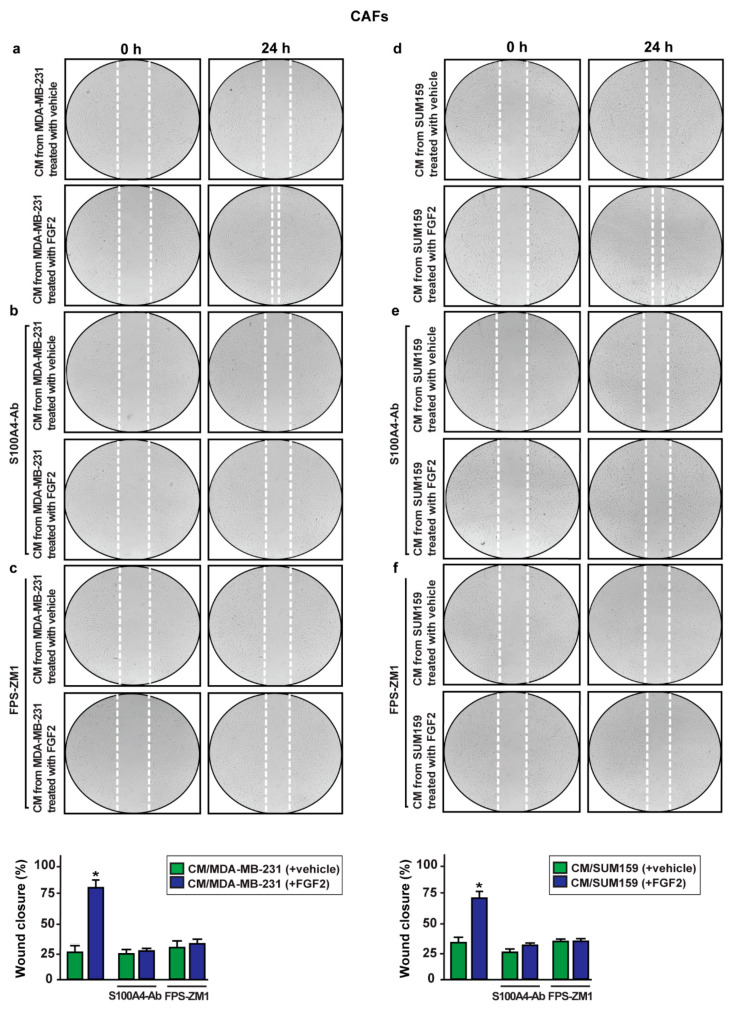
The paracrine action of the S100A4/RAGE axis promotes the migration of CAFs. (**a**,**d**) The migration of CAFs promoted by conditioned medium (CM) collected from MDA-MB-231 and SUM159 cells treated for 8 h with 25 nM FGF2 was no longer evident using CM collected from MDA-MB-231 and SUM159 cells treated for 8 h with vehicle and 25 nM FGF2 but immunodepleted for S100A4 by incubation with a mouse monoclonal antibody against S100A4 (S100A4-Ab) (**b**,**e**) or using 2 μM RAGE antagonist FPS-ZM1 (**c**,**f**). Images were acquired at 0 and 24 h after scratching. Quantification of cell migration was expressed as % of wound closure. Data shown are the mean ± SD of three independent experiments performed in triplicate. (*) indicates *p* < 0.05.

In addition, the migration of CAFs cultured in conditioned medium collected from MDA-MB-231 and SUM159 cells exposed to FGF2, was prevented immunodepleting S100A4 and using the RAGE inhibitor FPS-ZM1 (Figure 11). Altogether, these results show that the S100A4 release by FGF2-stimulated TNBC cells may elicit a paracrine action via RAGE toward the migration of CAFs.

## 3. Discussion

In the present study, we have provided novel findings regarding the role of the FGF2/FGFR1 transduction pathway on the regulation of S100A4 in TNBC cells. Querying the public TCGA dataset, we first ascertained that the expression of S100A4 is higher in TNBC with respect to non-TNBC patients and correlated with the levels of FGF2 in TNBC samples. Then, we demonstrated that FGF2 induces the upregulation and release of S100A4 through the FGFR1–ERK1/2–AKT–c-Rel transduction pathway in TNBC cells. Focusing on the functional liaison occurring between breast tumor cells and components of the surrounding stroma, we next assessed that S100A4 secreted by TNBC cells upon FGF2 treatment via RAGE endothelial tube formation and migratory responses in CAFs. Hence, our data extend the current knowledge on the transduction network activated by the FGF2/FGFR1 axis toward TNBC aggressiveness.

Great efforts have been recently addressed to better understand the role of the tumor microenvironment (TME) in cancer progression and the failure of antitumor therapies [71,72,73]. The TME is characterized by a multifaceted interplay occurring between the epithelial cancer cells and the neighboring non-cancerous stromal compartment that encompasses various cell types, including CAFs, endothelial, immune cells, and adipocytes [27]. In this context, it has been demonstrated that the release by either cancer cells or components of tumor stroma of various effectors such as growth factors, cytokines, chemokines, and others may promote the acquisition of aggressive and metastatic phenotypes [74,75]. For instance, it has been suggested that FGF2 may act as an important tumor-promoting factor [59], in accordance with data showing that its levels are elevated in TNBC cells and in plasma samples of TNBC and other types of tumors [25,76,77,78]. In addition, the upregulation and secretion of FGF2 have been shown to stimulate, in an autocrine and paracrine manner, the FGFR-mediated signaling toward cell proliferation, migration, and metastasis [59,79,80].

Despite the efforts made in recent years, metastatic evolution remains the primary cause of death in breast cancer patients [81]. Therefore, the identification of metastatic markers is a challenge for better monitoring aggressive tumor features in order to improve the survival rates of patients. In this regard, S100A4 (also known as metastasin or metastasis-associated protein) may be included among the predictive molecular biomarkers of cancer progression [36]. The S100A4 gene was originally isolated and characterized from metastatic cells and then associated to tumor metastasis [82,83]. In normal tissue, S100A4 was predominantly detected in the nervous system and connected to the neuronal plasticity in pathophysiological conditions [84,85]. S100A4 expression is regulated by epigenetic mechanisms and a complex signal transduction network [51,86,87]. Moreover, the expression of S100A4 can be boosted by diverse stimuli, including growth factors, cytokines, chemokines, and hypoxia in diverse types of cancers [50,51,52,53,54,88]. In this vein, our results have shown that FGF2 is involved in the regulation of S100A4 in TNBC cells. In particular, we have determined that the FGF2/FGFR1 axis induces the transcriptional activation of S100A4 in a NF-kB-dependent manner in TNBC cells. NF-kB is a protein complex that encompasses a family of Rel-like domain dimeric transcription factors exhibiting both shared and distinct biological functions [89]. Upon its shuttling from the cytoplasm to the nucleus, NF-kB interacts with specific DNA binding regions leading to the transcription of various genes [89]. Of note, we found that FGF2 induces the nuclear accumulation of c-Rel and its recruitment within the S100A4 promoter region toward the upregulation of S100A4 expression and secretion. These findings add novel insights to previous studies suggesting that S100A4 plays a role in the epithelial mesenchymal transition (EMT) process and in the pre-metastatic niche formation and is correlated with high tumor grade and poor survival of cancer patients [33,56,57,90,91].

The S100A4 protein, as a typical member of the S100 family, exerts both intracellular and extracellular activities [92]. Intracellularly, S100A4 binds to several protein targets involved in the regulation of cytoskeletal dynamic, cell adhesion, and the apoptotic process [32,93,94]. As extracellular protein released by both tumor or stroma cells, S100A4 acts in either an autocrine or paracrine manner by binding to its cognate receptor RAGE, thereby triggering cell migration, invasion, and angiogenic responses [37,38,88,95,96,97]. Notably, in accordance with these observations, we assessed that the inhibition of RAGE abrogates the paracrine action elicited by S100A4 toward angiogenesis and the migration of CAFs.

Collectively, our results indicate that S100A4 may be considered as a novel target of FGF2/FGFR1 transduction pathway in TNBC cells. Although further studies are warranted to deeply elucidate the peculiar oncogenic action of S100A4/RAGE, our findings may pave the way for more comprehensive therapeutic strategies aiming to halt TNBC aggressiveness.

## 4. Materials and Methods

### 4.1. Bioinformatics Analyses

In silico gene expression analyses were performed on R Studio (version 3.6.1) using the publicly available information of the Invasive Breast Cancer Cohort of The Cancer Genome Atlas (TCGA) project [98]. The clinical information and mRNA expression data (RNA Seq V2 RSEM) of patients were retrieved from UCSC Xena (https://xenabrowser.net/) on the 1st December 2020. The breast cancer samples of the TCGA cohort (n. 1247) were filtered by “sample type” in order to exclusively obtain the information of primary tumor tissues (n. 1104). Thereafter, patients were classified on the basis of the presence or absence of the estrogen receptor (ER), progesterone receptor (PR), and human epidermal growth factor receptor 2 (HER2). Gene expression and clinical information were also filtered for missing values, obtaining 650 non-TNBC and 123 TNBC patients. The Pearson correlation coefficient (r-value) was calculated using the R cor.test() function and setting the method as “Pearson”, and the statistical analysis was performed using the t-test. Box plots and scatter plots were performed with the R tidyverse package.

### 4.2. Reagents

We purchased PD173074 from Selleckchem (DBA, Milan, Italy), PD98059 from Calbiochem (DBA, Milan, Italy), Wortmannin (WM) and FPS-ZM1 from Sigma-Aldrich (Milan, Italy), and recombinant human fibroblast growth factor (FGF2) 100-18B from PEPROTECH (SIAL, Rome, Italy). All the aforementioned compounds were solubilized in dimethyl sulfoxide (DMSO) except for FGF2, which was dissolved in aqueous buffer (0.1% BSA).

### 4.3. Cell Cultures

The TNBC MDA-MB-231 cells were obtained from the ATCC (Manassas, VA, USA). The TNBC SUM159 cells were kindly provided by Dr. W.T. Khaled, University of Cambridge, UK. MDA-MB 231 cells were maintained in DMEM/F12 (Dulbecco’s modified Eagle’s medium) with phenol red, supplemented with 5% fetal bovine serum (FBS) and 1% penicillin/streptomycin (Thermo Fisher Scientific, Monza, Italy), while SUM159 cells were maintained in DMEM/F12 (Dulbecco’s modified Eagle’s medium) with phenol red and supplemented with 5% FBS, 5 μg/mL of insulin (Sigma-Aldrich, Milan, Italy), 1 μg/mL of hydrocortisone (Sigma-Aldrich, Milan, Italy), and 1% of penicillin/streptomycin (Thermo Fisher Scientific, Monza, Italy). Human umbilical vein endothelial cells (HUVECs), kindly provided by Dr. Caruso, University of Brescia, Italy, were seeded on collagen-coated flasks (Sigma-Aldrich, Milan, Italy) and cultured in endothelial growth medium (EGM) (Lonza, Milan, Italy) supplemented with 5% FBS (Lonza, Milan, Italy). Cells were used less than 6 months after resuscitation and mycoplasma negativity was tested monthly. Cancer-associated fibroblasts (CAFs) were isolated, cultured, and characterized as previously described [99] from 10 invasive mammary ductal carcinomas and pooled for the subsequent studies. Briefly, specimens were cut into small pieces (1–2 mm diameter), placed in digestion solution (400 IU collagenase, 100 IU hyaluronidase and 10% FBS, containing antibiotics and antimycotics solution), and incubated overnight at 37 °C. Cells were then separated by differential centrifugation at 90× *g* for 2 min. The supernatant containing fibroblasts were centrifuged at 485× *g* for 8 min, the obtained pellet was suspended in fibroblasts growth medium (Medium 199 and Ham’s F12 mixed 1:1 and supplemented with 10% FBS and 1% penicillin) (Thermo Fisher Scientific, Monza, Italy) and cultured at 37 °C, 5% CO_2_. CAFs were then expanded onto two 15 cm Petri dishes and stored as cells passaged for two to three population doublings within a total 7 to 10 days after tissue dissociation. Primary cell cultures of breast fibroblasts were characterized by immunofluorescence. In particular, cells were incubated with human anti-vimentin (V9, sc-6260) and human anti-cytokeratin 14 (LL001 sc-53,253), obtained from Santa Cruz Biotechnology (DBA, Milan, Italy) (data not shown). To characterize fibroblast activation, we used anti-fibroblast-activated protein α (FAPα) antibody (H-56; Santa Cruz Biotechnology, DBA, Milan, Italy) (data not shown). All cell lines were grown in a 37 °C incubator with 5% CO_2_. Cells were switched to medium without serum and phenol red 24 h before treatments to be processed for immunoblot and real-time PCR assays.

### 4.4. Gene Expression Studies

Total RNA was extracted, and cDNA was synthesized by reverse transcription as previously described [100]. The expression of selected genes was analyzed by real-time PCR using platform Quant Studio7 Flex Real-Time PCR System (Thermo Fisher Scientific, Monza, Italy). Gene-specific primers were designed using Primer Express version 2.0 software (Applied Biosystems) and sequences are as follows: 5′-GCAAAGAGGGTGACAAGTTC-3′ (S100A4 Fwd), 5′-TCTGGGAAGCCTTCAAAGAAT-3′ (S100A4 Rev), and 5′-AAGCCACCCCACTTCTCTCTAA-3′ (ACTB Fwd) and 5′-CACCTCCCCTGTGTGGACTT-3′ (ACTB Rev). Assays were performed in triplicate, and the results were normalized with control mRNA levels of actin beta (ACTB) and relative mRNA levels were calculated using the ΔΔCt method, comparing to the control group.

### 4.5. Gene Silencing Experiments and Luciferase Assays

Cells were plated onto 10 cm dishes and transfected using Lipofectamine LTX (Thermo Fisher Scientific, Monza, Italy) with a control vector (shRNA) and a specific shRNA sequence for c-Rel. c-Rel shRNA sequence (shc-Rel) was obtained from TRC consortium (TRCN0000039986) and cloned, as previously described [101], in the piggyBac transposon vector (PB-H1-shRNA-GFP), which was kindly provided by Dr. W.T. Khaled, University of Cambridge, UK. Two days after transfection, cells were selected in a medium containing G418 (200 μg/mL) (Sigma-Aldrich, Milan, Italy) for 5 days, and the G418-resistant colonies were picked and expanded in regular medium. Then, immunoblots for c-Rel protein were performed to evaluate the efficiency of the c-Rel silencing. The S100A4 promoter luciferase constructs pGl4.10 S100A4 (−632/+1010) and the mutant pGl4.10 S100A4 (+58/+1010) were a kind gift from Prof. K. L. O’Connor, Department of Molecular and Cellular Biochemistry, University of Kentucky, Lexington, KY (USA) [102]. The *Renilla* luciferase expression vector pRL-TK (Promega, Milan, Italy) was used as internal transfection control. Cells (1 × 10^5^) were plated into 24-well dishes with 500 µL/well culture medium containing 5% FBS. Cell medium was replaced on the day of transfection with serum-free medium, and transfection was performed using X-treme GENE 9 DNA Transfection Reagent as recommended by the manufacturer (Roche Diagnostics, Merck Life Science) with a mixture containing 0.5 µg of each reporter plasmid and 5 ng of pRL-TK. After 8 h, cells were treated with FGF2 alone and in combination with the FGFR1 inhibitor PD173074, the MEK inhibitor PD98059, or PI3K inhibitor Wortmannin and incubated for 18 h. Luciferase activity was then measured using the Dual Luciferase Kit (Promega, Milan, Italy) according to the manufacturer’s recommendations. Firefly luciferase activity was normalized to the internal transfection control provided by the *Renilla* luciferase activity. The normalized relative light unit values obtained from cells treated with vehicle (-) were set as 1-fold induction upon which the activity induced by treatments was calculated.

### 4.6. CRISPR/Cas9-Mediated FGFR1 Knockout

The MDA-MB-231 and SUM159 FGFR1 knockout (KO) cells were generated and characterized as previously described [79]. Briefly, short guide RNA (sgRNA) sequence targeting human FGFR1 was designed using the E-CRISP sgRNA Designer (http://www.e-crisp.org/E-CRISP/, accessed on 23 October 2017) and cloned into the pSpCas9 (BB)-2A-Puro (PX459) vector (a kind gift from Dr. W.T. Khaled, University of Cambridge, Cambridge, UK) according to the protocol described in Ran et al. [103]. The FGFR1 sgRNA sequence is as follows: 5′-CGGCCTAGCGGTGCAGAGTG-3’ (sgFGFR1). Then, the plasmid with sgRNA was transiently transfected into MDA-MB-231 and SUM159 cells using Lipofectamine LTX (Thermo Fisher Scientific, Monza, Italy). Two days after transfection, the cells were selected in a medium containing 1 µg/mL puromycin dihydrochloride (Sigma-Aldrich, Milan, Italy). After puromycin selection, the puromycin-resistant colonies were picked and expanded in regular growth medium. Then, immunoblots for FGFR1 protein were performed to evaluate the efficiency of FGFR1 KO.

### 4.7. Western Blot Analysis

Cells were processed to obtain protein lysates for Western blot analysis as previously described [104]. Primary antibodies were S100A4 (1C4) (Novus, DBA, Milan, Italy), FGFR1 (#9740), p-FGFR1 (#3476), p-AKT (Ser473) (D9E), and c-Rel (#4727) (Cell Signaling Technology, Euroclone Milan, Italy); p-ERK1/2 (E-4), ERK2 (C-14), AKT/1/2/3 (H-136) and β-actin (AC-15) (Santa Cruz Biotechnology, DBA, Milan, Italy). Proteins were detected using horseradish peroxidase-linked secondary antibodies (Biorad, Milan, Italy) and signals revealed using the chemiluminescent substrate for Western blotting Westar Nova 2.0 (Cyanagen, Biogenerica, Catania, Italy). For nuclear extracts, cells were lysed using 300 μL of cytosolic buffer (50 mM HEPES pH 7.5, 150 mM NaCl, 1% Triton X-100, 1.5 mM MgCl_2_, 1 mM EGTA, pH 7.5, 10% glycerol) with protease inhibitors (1.7 mg/mL aprotinin, 1 mg/mL leupeptin, 200 mmol/liter phenylmethylsulfonyl fluoride, 200 mmol/liter sodium orthovanadate and 100 mmol/liter sodium fluoride). Following centrifugation (14,000× *g*, 4 °C, 10 min), the supernatant was referred to as cytosolic fraction, and the pellet containing nuclei was resuspended in high salt buffer (20 mM HEPES pH 7.9, 25% [*v*:*v*] glycerol, 420 mM NaCl, 1.5 mM MgCl_2_, 0.2 mM EDTA, and protease inhibitors). For the extraction of nuclear proteins, the obtained solution was vortexed thoroughly, incubated overnight with agitation, and centrifuged at 14,000× *g*, 4 °C, for 10 min. Equal amounts of the collected supernatants, which represent the previously extracted nuclear fraction and cytosolic fraction, were then electrophoresed by SDS-PAGE on 10% gels and Western blot analysis was performed as described above. The purity of fractions was demonstrated immunoblotting with analysis of the cytosolic and nuclear marker proteins β-actin (AC-15) and lamin B1 (B-10) (Santa Cruz Biotechnology, DBA, Milan, Italy), respectively.

### 4.8. Immunofluorescence Microscopy

Cultured cells grown to 50% confluency on coverslips were serum-deprived for 24 h and then treated for 2 h with FGF2 alone and in combination with the FGFR1 inhibitor PD173074, the MEK inhibitor PD98059, or PI3K inhibitor Wortmannin, as indicated. Thereafter, cells were fixed in 4% paraformaldehyde, permeabilized with 0.2% Triton X-100, washed 3 times with PBS and incubated overnight with a rabbit primary antibody against c-Rel (#4727) (Cell Signaling, Euroclone, Milan, Italy). After incubation, the slides were extensively washed with PBS, probed with Alexa Fluor 594 goat anti-rabbit immunoglobulin G (IgG) (1:300, Thermo Fisher Scientific, Monza, Italy) and 4,6-diamidino-2-phenylindole dihydrochloride (DAPI) (1:1000; Sigma-Aldrich, Milan, Italy). Then, the slides were imaged on the Cytation 3 Cell Imaging Multimode reader (BioTek, AHSI, Milan Italy) and analyzed using the software Gen5 (BioTek, AHSI, Milan Italy).

### 4.9. Chromatin Immunoprecipitation (ChIP) Assay

Cells grown in 10 cm plates were shifted for 24 h to medium lacking serum, then treated with vehicle and FGF2 for 2 h, and then cross-linked with 1% formaldehyde and sonicated. Supernatants were immuno-cleared with salmon DNA/protein A-agarose (Merck Life Science, Darmstadt, Germany) and immunoprecipitated with anti-c-Rel antibody or nonspecific IgG (Santa Cruz Biotechnology, DBA, Milan, Italy). Pellets were washed, eluted with a buffer comprising 1% SDS and 0.1 mol/L NaHCO_3_, and digested with proteinase K. DNA was obtained by phenol/chloroform extractions and precipitated with ethanol. The yield of target region DNA in each sample after ChIP was analyzed by real-time PCR using platform Quant Studio7 Flex Real-Time PCR System (Thermo Fisher Scientific, Monza, Italy). The primers used to amplify a region containing an NF-kB site located into the S100A4 promoter sequence were: 5′-GCAAATGTTCACTGCCCAGA-3′ (Fwd) and 5′-ATCACATCCAGGGCCTTCTC-3′ (Rev). Real-time PCR data were normalized with respect to unprocessed lysates (Input) and the results were reported as fold changes with respect to nonspecific IgG.

### 4.10. Conditioned Medium

MDA-MB-231 and SUM159 cells were cultured in regular growth medium, then washed twice with PBS and switched to medium without serum for 24 h. Next, MDA-MB-231 and SUM159 cells were treated for 6 h with FGF2, as indicated. Culture medium was then removed, then cells were washed twice with PBS in order to eliminate any FGF2 residue and cultured for additional 8 h with fresh serum-free medium. Thereafter, the supernatants were collected, centrifuged at 3500 rpm for 5 min to remove cell debris, and used as conditioned medium in the appropriate experiments.

### 4.11. Acetone Precipitation of Proteins

Protein precipitation from conditioned medium derived from MDA-MB-231 and SUM159 cells was carried out using the precipitation method with acetone [105,106]. Briefly, four volumes of ice-cold acetone (Sigma-Aldrich, Milan, Italy) were added to one volume of sample. The mixture was vortexed and incubated at –20 °C overnight. This was followed by centrifugation at 10,000× *g* for 15 min at 4 °C. Afterwards, the supernatant was discarded, the pellet was air dried, then it was dissolved in 2× Laemmli buffer and used in the appropriate experiments. In Western blot analysis, the protein loading of conditioned medium samples was checked by Ponceau red staining (0.1% Ponceau S (*w*/*v*) in 5% acetic acid) of the blotted membranes.

### 4.12. S100A4-Immunodepleted Conditioned Medium

To deplete S100A4, conditioned medium was collected from MDA-MB-231 and SUM159 cells that were treated as indicated and cleared of cells by centrifugation. Thereafter, according to the previously reported protein immunodepletion protocol [107,108,109], protein A/G-agarose beads were incubated with anti-S100A4 or IgG antibodies for 3 h at 4 °C. Then, antibody–bead complexes were incubated with MDA-MB-231 and SUM159 cell-derived conditioned medium overnight and centrifuged. S100A4 immunodepletion was verified by immunoblotting and the S100A4-immunodepleted medium was used in endothelial tube formation and scratch assays, as indicated.

### 4.13. Tube Formation Assay

The day before the experiment, confluent HUVECs were starved overnight at 37 °C in serum-free medium. Growth factor-reduced Matrigel^®^ (Cultrex, Trevigen Inc., Gaithersburg, MD, USA) was thawed overnight at 4 °C on ice, plated on the bottom of pre-chilled 96-well plates, and left at 37 °C for 1 h for gelification. Starved HUVECs were collected by enzymatic detachment (0.25% trypsin-EDTA solution, Thermo Fisher Scientific, Monza, Italy), counted, and resuspended in conditioned medium collected from MDA-MB-231 or SUM159 cells treated as indicated. Then, 10,000 cells/well were seeded on Matrigel and incubated at 37 °C. Tube formation was observed starting from 6 h after cell seeding and quantified using WCIF ImageJ software.

### 4.14. Scratch Assay

CAFs were seeded into 12-well plates and were allowed to grow in regular growth medium until they were 70–80% confluent. Next, cells were switched in medium without serum, and after 24 h, a p200 pipette tip was used to create a scratch of the cell monolayer. Cells were washed twice with PBS and then incubated at 37 °C with conditioned medium collected from MDA-MB-231 or SUM159 cells for 24 h, as indicated. Pictures were photographed at 0 and 24 h after scratching using inverted phase contrast microscope (5× magnification). The rate of cell migration was measured by quantifying the % of wound closure area, determined using the software WCIF ImageJ, according to the following formula:

% of wound closure = [(At = 0 h) – (At = Δ h)/(At = 0 h)] × 100%

### 4.15. Statistical Analysis

Data were analyzed by one-way ANOVA with Dunnett’s multiple comparisons where applicable, using GraphPad Prism version 6.01 (GraphPad Software, Inc., San Diego, CA, USA). (*) *p* < 0.05 was considered statistically significant.

## Data Availability

The data presented in this study are available on request from the corresponding author. Publicly available dataset was analyzed in this study. This data can be found here: http://www.cbioportal.org, accessed on 1 December 2020.

## Supplementary Materials

The following are available online at https://www.mdpi.com/article/10.3390/ijms22094720/s1. Figure S1: Efficiency of CRISPR/Cas9-mediated FGFR1 knockout (KO) in MDA-MB-231 and SUM159 cells; Figure S2: FGF2 induces the activation of FGFR1 signaling pathway in MDA-MB-231 and SUM159 cells; Figure S3: The activation of FGFR1–ERK1/2–AKT signaling pathway prompts c-Rel nuclear accumulation in MDA-MB-231 cells; Figure S4: The activation of FGFR1–ERK1/2–AKT signaling pathway triggers c-Rel nuclear accumulation in SUM159 cells; Figure S5: Evaluation of c-Rel protein expression in MDA-MB-231 and SUM159 cells; Figure S6: Efficiency of S100A4 immunodepletion in conditioned medium (CM) collected from MDA-MB-231 and SUM159 cells.
