# Supplementary material for: S100A4 Is Involved in Stimulatory Effects Elicited by the FGF2/FGFR1 Signaling Pathway in Triple-Negative Breast Cancer (TNBC) Cells
Source: Int J Mol Sci. 2021 Apr 29;22(9):4720. doi: 10.3390/ijms22094720 (PMC8124532; doi:10.3390/ijms22094720)
Supplement: Supplementary file 1 [file ijms-22-04720-s001.zip › ijms-1182005-supplementary.pdf]

International Journal of
Molecular Sciences

MDPI

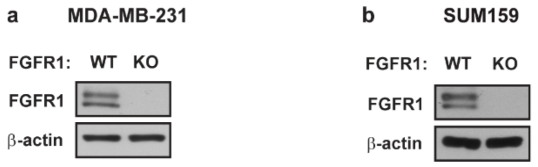

Figure S1. Efficiency of CRISPR/Cas9-mediated FGFR1 knockout (KO) in MDA-MB-231 and SUM159 cells. (**a**,**b**) Immunoblots of lysates generated from FGFR1 (WT) and FGFR1 (KO) MDA-MB-231 and SUM159 cells. β-Actin served as loading control. Immunoblots shown are representative of three independent experiments.

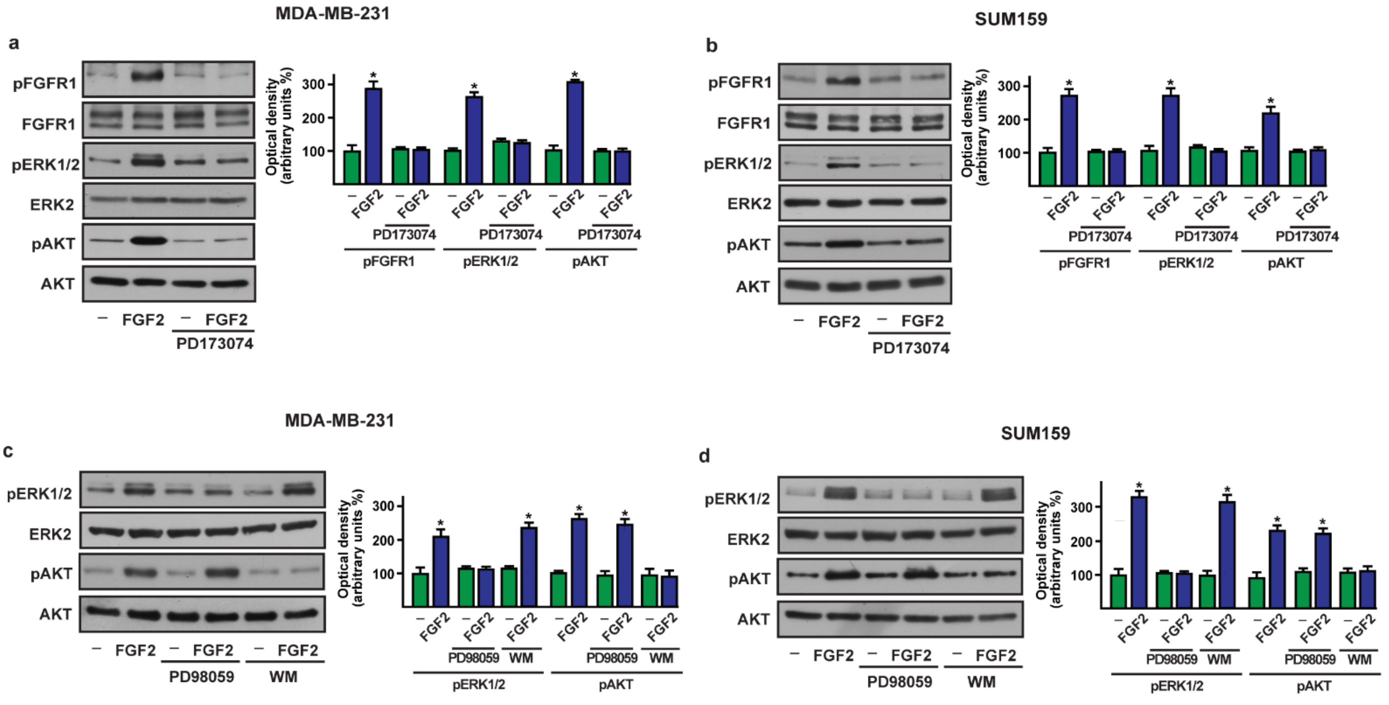

Figure S2. FGF2 induces the activation of FGFR1 signaling pathway in MDA-MB-231 and SUM159 cells. (a,b) Phosphorylation of FGFR1, ERK1/2, and AKT in MDA-MB-231 and SUM159 cells exposed for 1h to vehicle (-) and 25 nM FGF2 alone and in the presence of 1 μM FGFR1 inhibitor PD173074. **(c,d)** Phosphorylation of ERK1/2 and AKT in MDA-MB-231 and SUM159 cells exposed for 1 h to vehicle (-) and 25 nM FGF2 alone and in the presence of 1 μM MEK inhibitor PD98059 or 100 nM PI3K inhibitor Wortmannin (WM). FGFR1, ERK2, and AKT served as loading controls, as indicated. Side panels show densitometric analysis of the blots normalized to the loading control. Values represent the mean ± SD of three independent experiments. (*) indicates $p < 0.05$.

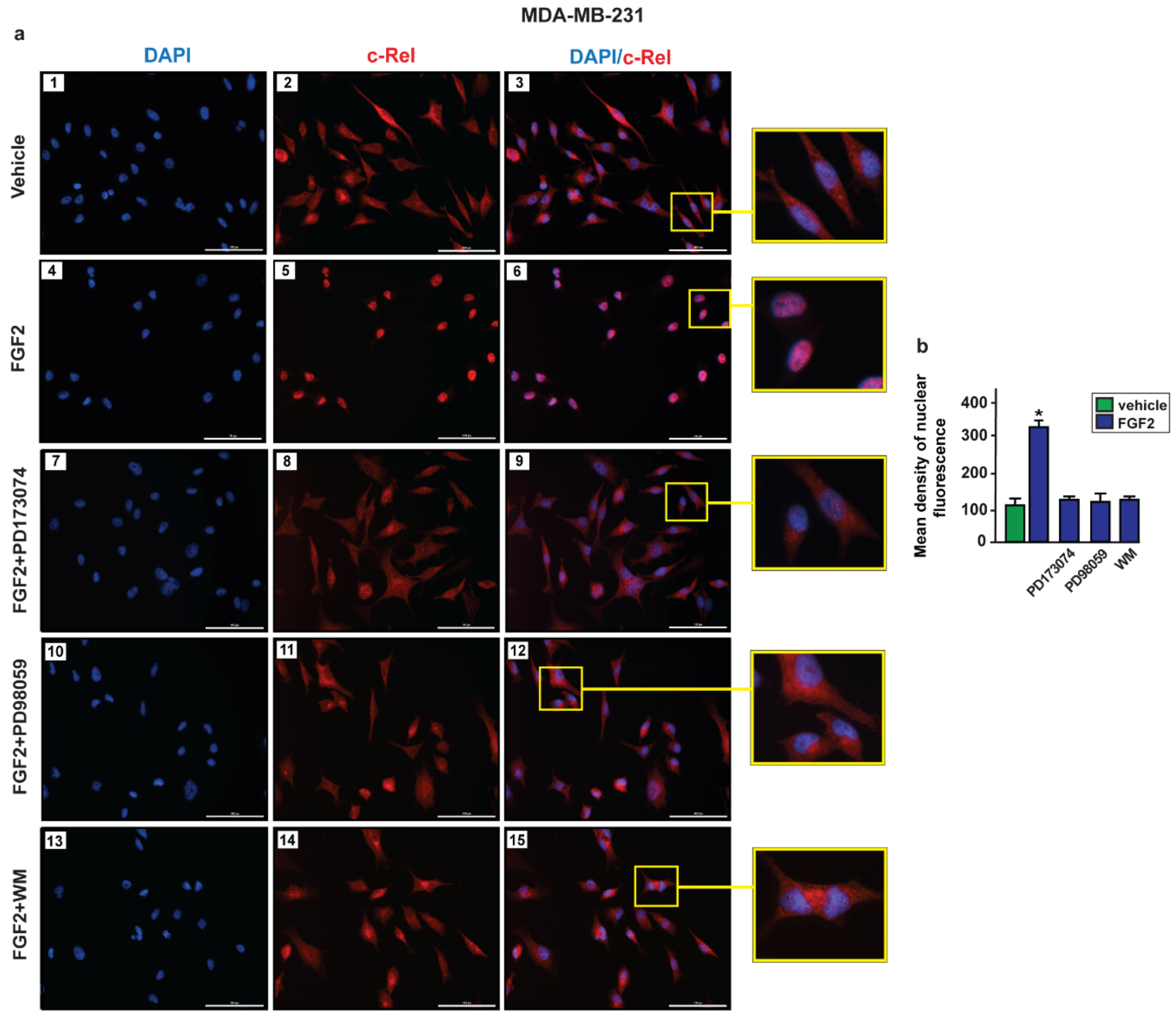

Figure S3. The activation of FGFR1-ERK1/2-AKT signaling pathway prompts c-Rel nuclear accumulation in MDA-MB-231 cells. (a) Immunofluorescence staining of c-Rel in MDA-MB-231 cells treated for 2 h with vehicle and 25 nM FGF2 alone (*panels 1–6*) and in the presence of 1 µM FGFR1 inhibitor PD173074 (*panels 7–9*), 10 µM MEK inhibitor PD98059 (*panels 10–12*), or 100 nM PI3K inhibitor Wortmannin (WM) (*panels 13–15*). c-Rel localization is shown in red, nuclei are stained by DAPI (blue), scale bar = 100 µm. Enlarged details are shown in the separate box. Images shown are representative of two independent experiments. (**b**) Quantification of nuclear c-Rel in cells treated with vehicle versus cells exposed to FGF2 alone and in combination with inhibitors reported above, as indicated. (*) indicates $p < 0.05$.

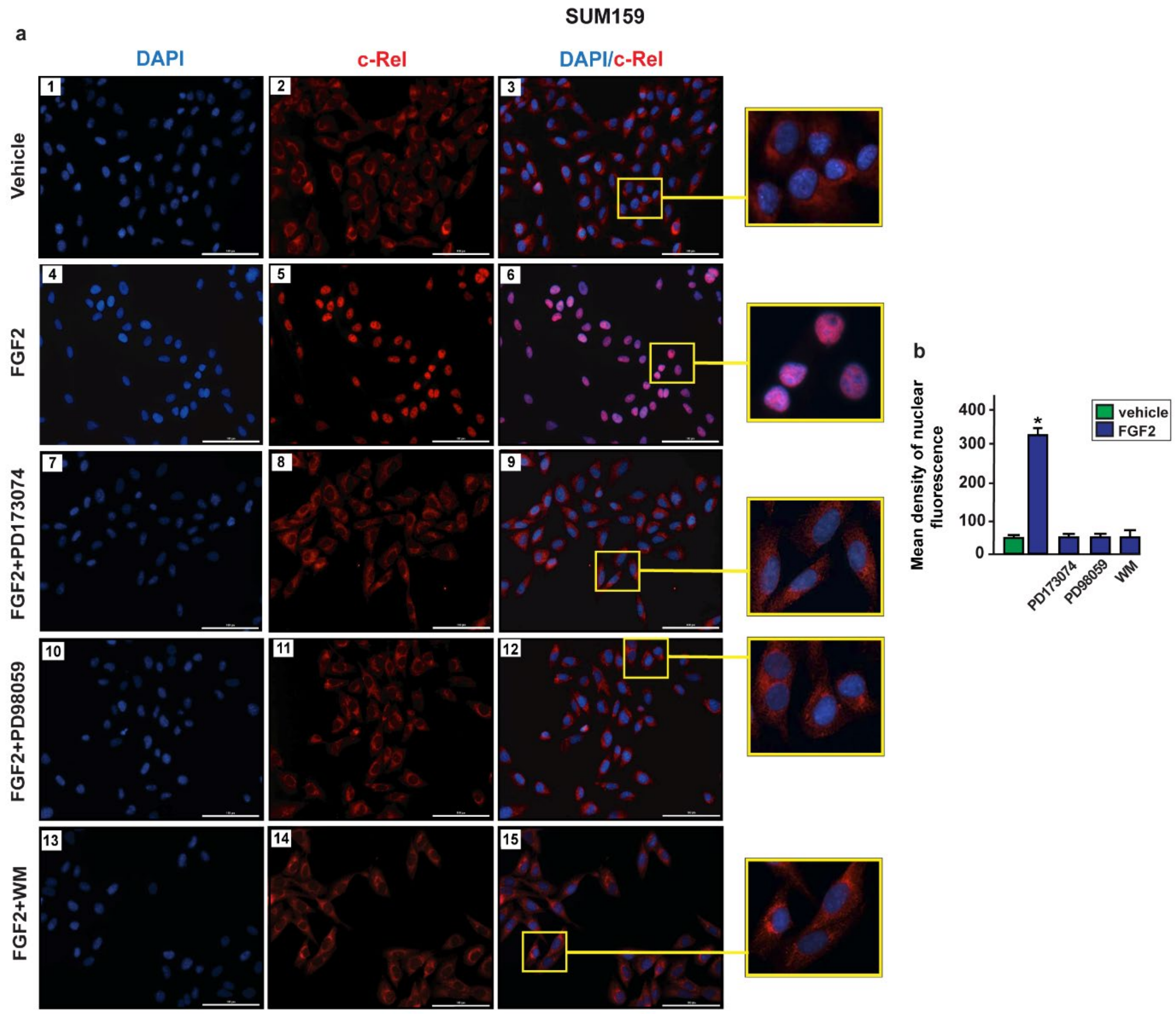

Figure S4. The activation of FGFR1-ERK1/2-AKT signaling pathway triggers c-Rel nuclear accumulation in SUM159 cells. (a) Immunofluorescence staining of c-Rel in SUM159 cells treated for 2 h with vehicle and 25 nM FGF2 alone (*panels 1–6*) and in the presence of 1 μM FGFR1 inhibitor PD173074 (*panels 7–9*), 10 μM MEK inhibitor PD98059 (*panels 10–12*), or 100 nM PI3K inhibitor Wortmannin (WM) (*panels 13–15*). c-Rel localization is shown in red, nuclei are stained by DAPI (blue), scale bar = 100 μm. Enlarged details are shown in the separate box. Images shown are representative of two independent experiments. (**b**) Quantification of nuclear c-Rel in cells treated with vehicle versus cells exposed to FGF2 alone and in combination with inhibitors reported above, as indicated. (*) indicates $p < 0.05$.

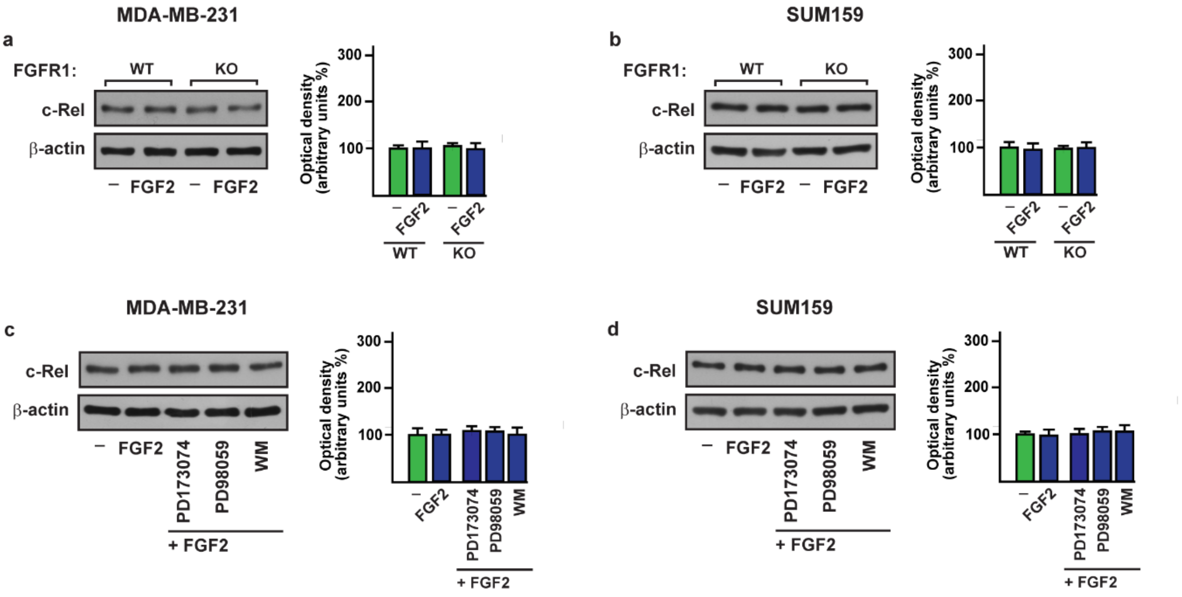

Figure S5. Evaluation of c-Rel protein expression in MDA-MB-231 and SUM159 cells. (a,b) c-Rel protein expression evaluated by immunoblotting in FGFR1 (WT) and FGFR1 (KO) MDA-MB-231 and SUM159 cells treated for 2 h with vehicle (-) and 25 nM FGF2, as indicated. **(c, d)** Immunoblots showing c-Rel protein expression in MDA-MB-231 and SUM159 cells exposed for 2 h to vehicle (-) and 25 nM FGF2 alone and in the presence 1 μM FGFR1 inhibitor PD173074, 1 μM MEK inhibitor PD98059, or 100 nM PI3K inhibitor Wortmannin (WM). β-Actin served as a loading control, side panels show densitometric analysis of the blots normalized to β-actin. Values represent the mean ± SD of three independent experiments.

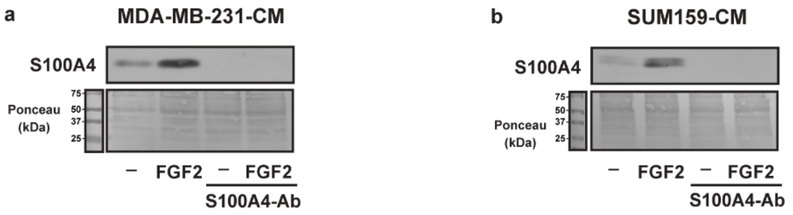

Figure S6. Efficiency of S100A4 immunodepletion in conditioned medium (CM) collected from MDA-MB-231 and SUM159 cells. (**a**,**b**) Evaluation by immunoblotting of S100A4 levels in conditioned medium (CM) derived from MDA-MB-231 and SUM159 cells treated for 8 h with vehicle (-) and 25 nM FGF2, and in CM collected from MDA-MB-231 and SUM159 cells treated for 8 h with vehicle (-) and 25 nM FGF2, in which S100A4 was immunodepleted by incubation with a mouse monoclonal specific antibody against S100A4 (S100A4-Ab). Ponceau red staining of the membrane was used as a loading control for the CM. Immunoblots shown are representative of three independent experiments.
